# UnAIDed Class Switching in Activated B-Cells Reveals Intrinsic Features of a Self-Cleaving IgH Locus

**DOI:** 10.3389/fimmu.2021.737427

**Published:** 2021-10-28

**Authors:** Iman Dalloul, Brice Laffleur, Zeinab Dalloul, Batoul Wehbi, Florence Jouan, Baptiste Brauge, Paco Derouault, Jeanne Moreau, Sven Kracker, Alain Fischer, Anne Durandy, Sandrine Le Noir, Michel Cogné

**Affiliations:** ^1^ Institut National de la Santé et de la Recherche Médicale (INSERM) U1262, Centre National de la Recherche Scientifique (CNRS) Unité Mixte de Recherche (UMR) 7276, Limoges University, Limoges, France; ^2^ Institut National de la Santé et de la Recherche Médicale (INSERM) U 1236, Rennes1 University, Rennes, France; ^3^ Centre Hospitalier Universitaire (CHU) Dupuytren, Limoges, France; ^4^ Institut National de la Santé et de la Recherche Médicale (INSERM) Unité Mixte de Recherche (UMR) 1163, Laboratory of Human Lympho-hematopoiesis, Imagine Institute, Université de Paris, Paris, France

**Keywords:** B lymphocyte, class switch DNA recombination (CSR), AICDA, immunoglobulin, class switch

## Abstract

Activation-induced deaminase (AID) is the major actor of immunoglobulin (Ig) gene diversification in germinal center B-cells. From its first description, it was considered as mandatory for class switch recombination (CSR), and this discovery initiated a long quest for all of the AID-interacting factors controlling its activity. The mechanisms focusing AID-mediated DNA lesions to given target sequences remain incompletely understood with regards the detailed characterization of optimal substrates in which cytidine deamination will lead to double strand breaks (DSBs) and chromosomal cleavage. In an effort to reconsider whether such CSR breaks absolutely require AID, we herein provide evidence, based on deep-sequencing approaches, showing that this dogma is not absolute in both human and mouse B lymphocytes. In activated B-cells from either AID-deficient mice or human AID-deficient patients, we report an intrinsic ability of the *IgH* locus to undergo “on-target” cleavage and subsequent synapsis of broken regions in conditions able to yield low-level CSR. DNA breaks occur in such conditions within the same repetitive S regions usually targeted by AID, but their repair follows a specific pathway with increased usage of microhomology-mediated repair. These data further demonstrate the role of AID machinery as not initiating *de novo* chromosomal cleavage but rather catalyzing a process which spontaneously initiates at low levels in an appropriately conformed *IgH* locus.

## Introduction

Germinal center B-cells actively undergo remodeling of their immunoglobulin (Ig) loci while being selected for antigen binding. This results in the emergence of cells carrying a B-cell receptor with higher affinity for antigen after somatic hypermutation (SHM) of rearranged Ig V(D)J genes and which undergo class switch recombination (CSR) of Ig heavy chain (*IgH*) constant (*C_H_*) genes. SHM and CSR are initiated by activation-induced deaminase (AID), a member of the AID/APOBEC family of enzymes deaminating cytidines into uridines. By initiating DNA lesions in the repetitive S regions that precede *C_H_* regions, AID is the key enzyme responsible for the CSR of *C_H_* genes (1, 2). In some conditions, it can also initiate complete deletion of the constant gene cluster and locus suicide recombination (3, 4).

The targeting of *Ig* genes by AID requires preexisting chromatin accessibility and transcription, which expose single-stranded DNA within transcription bubbles and R-loops. In such Ig target sequences, AID deamination is focused on WRC motifs (W = A/T, R = A/G) (5). Off-target lesions are also found at a much lower frequency in some non-Ig genes transcribed in B-cells and can eventually contribute to lymphomagenesis (6, 7).

The *C_H_* regions of Ig genes usually escape AID lesions while being transcribed by RNA polymerase II (RNAPII) in its elongating form (phosphorylated on the C-terminal domain at Ser2) and carrying the histone marks H4K20me1 and H3K36me3, which recruit histone acetyltransferases (8). In contrast, the S regions are enriched for hyperacetylated (Ac) H3K9 and trimethylated histone H3 on lysine 4 (H3K4me3), with local recruitment of histone deacetylases (8, 9). Sµ is additionally enriched in trimethylated histone H3 at lysine 9 (H3K9me3), which recruits KRAB domain-associated protein 1 and heterochromatin protein 1 (HP1) (10). On its main targets, i.e., V and S regions, AID interacts with stalled RNAPII phosphorylated at Ser5 and bound by the transcription elongation factor Spt5 (11). Additional RNAPII-associated factors (PAF) also help recruit AID (12). Both V and S regions are locations for paused RNAPII, but this is increased within S regions by the local abundance of DNA repeats, secondary structures, and R-loops where transcribed RNA remains associated with the DNA template strand (13). AID preferentially binds to structured DNA, notably G-quadruplex (G4) structures, with also a likely contribution of G4-rich transcripts in AID recruitment (14, 15). In mammals, dense G4 structures present on the non-template strand of S-regions promote the formation of R-loops, and pharmacological G4 ligands were shown to inhibit CSR (16). To access both strands of transcribed Ig genes *in vivo*, AID requires a prior activity of the RNA exosome complex, tethered to RNAPII by Spt5/Spt6 (17). Within R-loops, the RNA exosome removes RNA from the template strand, which provides equivalent accessibility of both DNA strands to cytosine deamination by AID and also participates in the correct conformation of the topologically associating domain (17, 18). The S regions have specific transcription patterns, with abundant antisense transcription and the presence of multiple alternate transcription start sites (19). The location of S regions within spliced introns is an additional prerequisite for CSR and might promote the interactions of AID and Spt5 with spliceosome-associated factors, such as CTNNBL1 (9, 20).

These features altogether engender an abundant occurrence of DNA lesions along extended domains of the target S regions, where staggered AID-initiated single-strand cleavages affecting either DNA strand are followed by DSBs. While such breaks are often considered as totally AID dependent, we wished to reconsider whether they could occur at low levels in the absence of AID.

## Materials and methods

### Patients

DNA from AID-deficient human patients included in this study included one tonsil DNA sample (patient P4) and DNA from three peripheral blood samples (patients P3, P5, and P6). Biallelic mutations within the AICDA coding sequence were identified in all patients. Patient P3 carried a nonsense W68X mutation at the beginning of the AICDA exon 3 (which encodes the catalytic domain of AID) on one allele and a complete deletion of exon 3 on the other allele. Patients P4 and P5 were family relatives, and both carried the same W68X nonsense mutation within exon 3 on one allele and a three-codon deletion affecting exon 3 on the other allele. These three patients have been described in detail, including for clinical manifestations and immune phenotype in the initial report from Revy et al. (2). Besides serum IgM at 1, 1.5, and 2.4 mg/ml, respectively, in patients P3, P4, and P5, those three patients had serum levels of class-switched IgG and IgA below the detection threshold, except for a low but detectable level (0.4 mg/ml) of serum IgG in patient P4 (2). Patient P6 is previously unpublished and carried a homozygous K22X nonsense mutation terminating the AID coding sequence within exon 2. The serum Ig levels in this patient were as follows: IgM, 0.65 mg/ml; IgG, 0.10 mg/ml; and IgA below 0.01 mg/ml. The AID alterations in patients are summarized in **Supplementary Figure S1**. Patients P3 and P6 are clearly affected on both alleles with loss-of-function (LOF) mutations severely truncating or deleting the catalytic domain. Patients P4 and P5 carry a clear LOF mutation on one allele, while it is debatable whether partial enzymatic activity could remain for the allele affected with the three-codon deletion.

Samples were obtained after receiving informed consent from the parents of patients.

### Mice

Our research was conducted under ethical agreement APAFIS no. APAFIS#16689-2018091017202113 v3. The wild-type (WT), homozygous RAG2-deficient (referred to as Rag^-/-^), and homozygous AID-deficient (referred to as AID ^-/-^) mice (a kind gift from Pr. T. Honjo) used for our experiments were maintained at 21–23°C with a 12-h light/dark cycle.

### Ovalbumin and Sheep Red Blood Cells

WT, Rag, and AID-deficient mice were injected intraperitoneally with an emulsion of 50% V/V complete Freund’s adjuvant (Sigma-Aldrich) and 1 mg/ml ovalbumin (Sigma-Aldrich). The mice were sacrificed at day 14.

The WT, Rag, and AID-deficient mice were injected intraperitoneally with 200 µl sheep red blood cells (SRBC) at day 0 and were boosted with 200 µl SRBC at day 7. The mice were sacrificed at day 17.

### Sample and Cell Preparations

Blood samples were recovered from WT, RAG, and AID-deficient mice at days 0, 7, and 14 after ovalbumin (OVA) immunization and at days 0, 7, and 17 after SRBC immunization with heparinized needles. Plasma samples were recovered by centrifugation and stored at -20°C until use.

Splenocytes were collected at sacrifice, red blood cells were lysed, and B-cells were isolated using EasySep™ Mouse B-cell Isolation Kit (Stem cell). B-cells were cultured for 4 days in RPMI containing 10% fetal calf serum with lipopolysaccharide (LPS) (1 µg/ml) (Invivogen) + IL-4 (20 ng/ml) (Peprotech). Supernatants were recovered and stored at -20°C until use.

For immunofluorescence, sections (18μm thick) of frozen spleen fixed with acetone, were labeled with fluorescent Abs (Jackson immunoresearch, Alexa 647 Goat Anti-Mouse IgG1, ref 115-605-205 and Alexa 488 Goat Anti- IgG2b, ref 115-545-207). Nuclei were stained with DAPI.

### Class-Specific ELISA

ELISA was performed on sera or supernatants from *in vitro* stimulated primary B-cells for the detection of various Ig classes and subclasses. Plates were coated overnight with monoclonal antibodies specific for IgM, IgA, IgG1, and total IgG (Southern Biotech). Anti-OVA-specific Abs produced *in vivo* after immunization were evaluated in sera by coating the plates with 10 μg/ml OVA. Sera or supernatants were added and incubated for 2 h at 37°C. After washing, alkaline phosphatase (AP) conjugates of goat anti-mouse IgM, IgG1, IgA, and total IgG (Southern Biotech) were incubated for 1 h at 37°C. Following washing and addition of AP substrate (Sigma), absorbance was measured at 405 nm. The specificity of the anti-sera used in ELISA for the identification of class-switched Ig was checked by verifying the absolute lack of signal yielded in the assays when using four different monoclonal mouse IgM clones [mouse IgM isotype control clone 11E10 (Southern Biotechnologies) and anti-ABO mouse IgM clones BHS17, AY144, and E11 (French National Blood Center)] as negative controls (**Supplementary Figure S2**). The specificity of class-switched Ig ELISA detection was further indicated by a polyclonal internal control, observing that serum from a naive mouse with spontaneously high IgM was not producing detectable IgG or IgA prior to immunization (**Supplementary Figure S2**).

### Amplification of Sμ/Sγ Junctions for Sequencing by CSRseq

DNA from SRBC immunized B-cells isolated from spleens of *WT* and AID-deficient mice was extracted using GenElute Mammalian Genomic DNA miniprep Kit (Sigma Aldrich). Murine Sμ/Sγ junctions were amplified in triplicate by nested PCR with 100 ng DNA (Phusion HF polymerase, BioLabs) using the following primers: Sµ Nest1 For (5′-AGAGACCTGCAGTTGAGGCC-3′) and Sγ consensus1 Rev (5′- TCAGGGAARTAVCCYTTGACCAGGCA-3′) for PCR1 Sμ/Sγ junctions and Sµ Nest2 For (5′-CCAGCCACAGTAATGACCCAG-3′) and Sγ consensus2 Rev (5′-CCARKGGATAGACHGATGGGG-3′) for PCR2.

Human Sμ/Sγ junctions were amplified as previously described (4). Each library was prepared using 200 ng of PCR2 product. Barcoded libraries with 200-bp read lengths were prepared using Ion Xpress plus Fragment Library Kit (Thermo Fisher Scientific) according to the instructions of the manufacturer. Each barcoded library was mixed in equal amounts and diluted to 100 pM. The libraries were run on chip 540 on the Ion S5 sequencer (Life Technologies). Data were analyzed using the CSReport software (21). This algorithm first aligns sequences with Sµ (set for identities higher than 90% and longer than 40 nucleotides) and explores identities to downstream S regions when the alignment with Sµ stops (again tracking identities higher than 90% and longer than 40 bp). The annotated IgH locus switch sequences considered for aligning mouse and human sequences are provided in **Supplementary Table S1**.

Quality controls for the whole CSRseq procedure process were done by processing non-lymphoid DNA samples (embryonic stem cell DNA for mouse assays and DNA from the human carcinoma cell line Hep2 for human assays). For both human and mouse assays, no CSR junction was obtained from such non-lymphoid samples, validating that the protocol safely identifies true CSR junctions from the template DNA and not PCR-built assemblies.

### Data Accessibility

Raw sequencing data and a table reporting processed data from the CSreport algorithm have been deposited on GEO.

## Results

We evaluated the AID dependence of B-cell responses using a colony of homozygous AID-deficient mice that had been bred for at least two generations.

**Figure 1:**
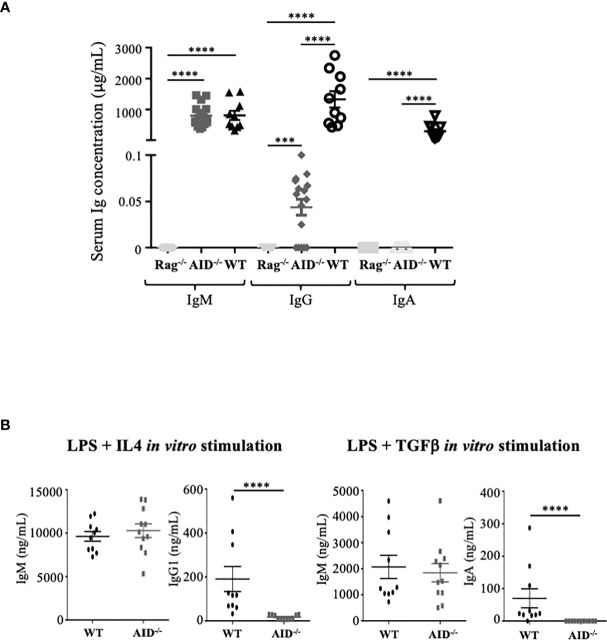
Ig secretion by activation-induced deaminase (AID)-deficient mice. **(A)** Basal serum level of Ig in non-immunized mice. **(B)** *In vitro* secretion of Ig in B-cells from wild-type (WT) and AID-deficient mice stimulated for 4 days with lipopolysaccharide and IL-4 (left) or with LPS+TGFβ (right). The supernatants were quantified by ELISA for IgM, IgG1, and IgA. The data represent mean concentrations ± SEM from two independent experiments with at least three WT and five AID-deficient mice. Mann–Whitney test was used for significance. ****p < 0.0001.

Circulating Ig levels were quantified in blood from 8–10-week-old animals. Total Ig levels from AID-deficient mice were compared to those from either wild-type or RAG2-/- immunodeficient mice. Circulating IgM in mice bred in specific and opportunistic-free conditions did not significantly differ from wild-type controls, in agreement with the lack of overt hyper-IgM previously documented by Honjo and colleagues in 10-week-old AID-deficient mice (22). By contrast, the total IgA level was below the detection threshold, while class-switched IgG was very low, with a mean ± standard deviation of 55 ± 35 ng/ml, but still clearly detectable (**Figure 1A**).

A similar profile was obtained when evaluating Ig production *in vitro* in supernatants of B-cells activated with either LPS+TGFβ (**Figure 1B**, right panel) or LPS+IL4 (**Figure 1B**, left panel), which respectively yielded no IgA but a low level of IgG1, together with IgM secreted in amounts similar to WT B-cells. Basal IgG production thus remains possible both *in vivo* in mice and *in vitro* in stimulated B-cells, in conditions of complete AID deficiency.

**Figure 2:**
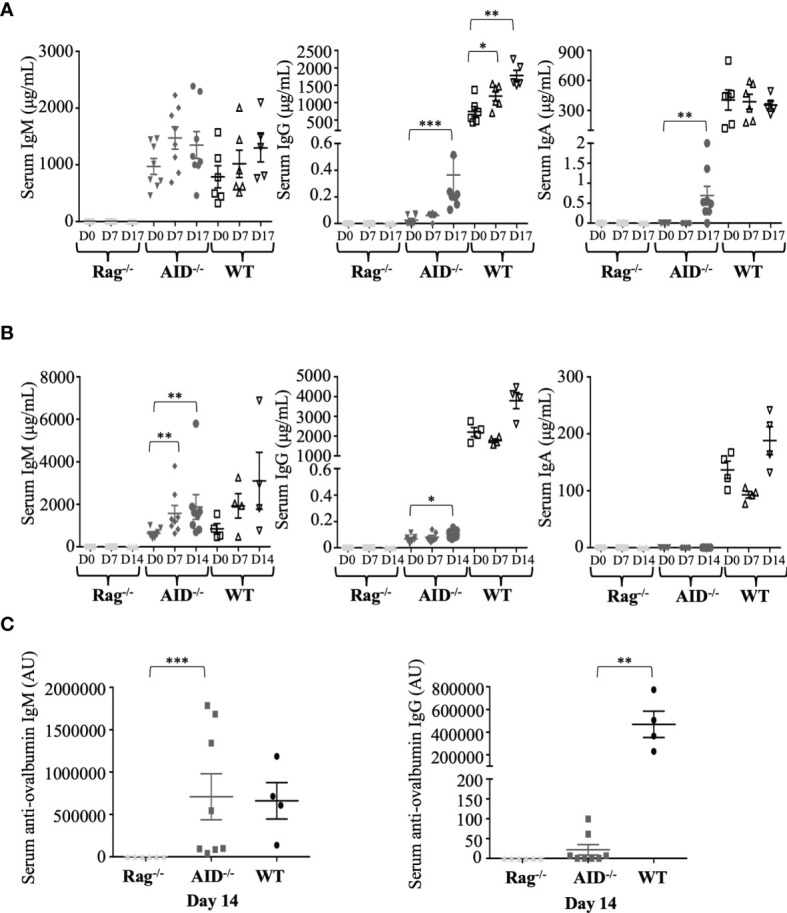
Dynamic induction of Ig production after mouse immunization. **(A)** Total IgM, IgG, and IgA concentrations in serum from immunized mice were evaluated by ELISA before and at 17 days after immunization with sheep red blood cells (samples were taken at days 0, 7, and 17, with booster immunization at day 7). **(B)** Total IgM, G, and A concentrations in serum from immunized mice were evaluated by ELISA before and at 14 days after ovalbumin (OVA) immunization at days 0, 7, and 14. **(C)** Ag-specific IgG1 and IgM antibodies were evaluated by ELISA after 14 days of intraperitoneal OVA immunization. The data in **(A)** represent mean concentrations ± SEM from five RAG-deficient, eight activation-induced deaminase (AID)-deficient, and six wild-type (WT) mice. Mann–Whitney test was used for significance. The data in **(B, C)** represent mean concentrations ± SEM from six RAG-deficient, eight AID-deficient mice, and four WT mice. Mann–Whitney test was used for significance. *p < 0.05; **p < 0.01; ***p < 0.001.

Since the functional role of Ig is to bind specific antigens as antibodies, we evaluated the dynamic process of the humoral response following immunization of AID-proficient compared to AID-deficient animals and to RAG-deficient mice. We monitored total IgM, IgG1, or IgA after immunization with the particulate Ag SRBC. In WT mice, total serum IgM and total serum IgA did not significantly vary from day 0 to 17 (**Figure 2A**, left panel), while at a much lower level, AID-deficient mice, by contrast, displayed about an eightfold increase in total IgG during the same time period, reaching a mean of 0.4 ± 0.38 μg/ml, and total IgA which was initially undetectable but finally reached a mean of 0.7 ± 0.65 μg/ml at day 17 (**Figure 2A**, middle and right panels). The validity of these evaluations of switched Ig classes, without cross-reaction with IgM, was further attested by the lack of correlation with total IgM levels (**Supplementary Figure S3**). As expected, all Ig levels remained undetectable in control RAG-deficient mice immunized in parallel.

Immune stimulation with a more strictly defined protein Ag, OVA, resulted in the same profile but with a weaker impact on total IgG levels and with no detectable induction of serum IgA (**Figure 2B**). The latter condition of OVA immunization yielded anti-OVA IgM at normal levels in AID-deficient mice but was also associated with anti-OVA IgG1 at low but significant levels, clearly above the background obtained in non-responding RAG-deficient mice (**Figure 2C**).

The switched Ig produced in low amounts in AID-deficient mice was thus not just bystander products secreted after random recombination events in B-cells but dynamically followed B-cell stimulation and included Ag-specific switched IgG after immunization.

**Figure 3:**
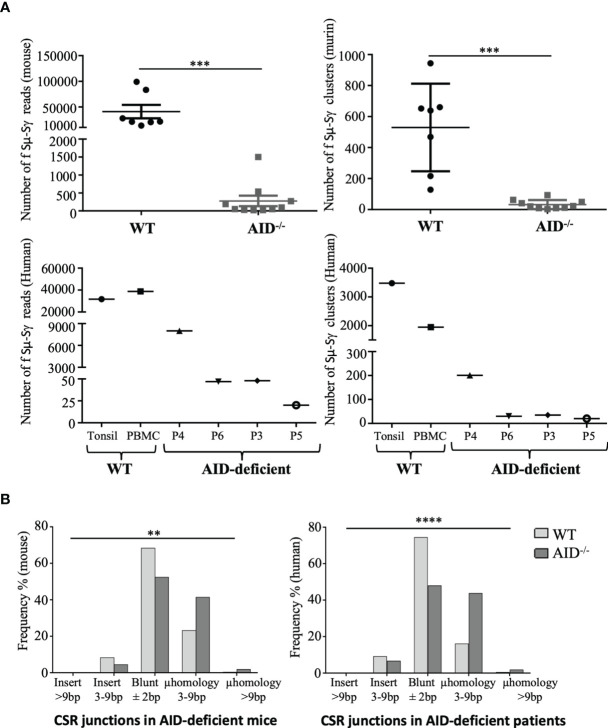
Class switch recombination (CSR) in murine and human activation-induced deaminase (AID)-deficient samples. **(A)** Sµ-Sγ murine CSR junctions were PCR-amplified and sequenced from spleen B-cells at 17 days after sheep red blood cell immunization, comparing mutant and control mice (top). Sµ-Sγ human CSR junctions were also PCR-amplified and sequenced from blood and tonsils from AID-deficient human samples (bottom). Numbers of independent reads including Sµ–Sγ junctions are shown on the left graph. Independent reads including the same CSR breakpoint were assembled and considered as clusters; numbers of independent clusters are shown on the right graph. Junction structures were analyzed using CSReport. **(B)** Structures of repaired junctions were analyzed depending on the mean number of inserted nucleotides and length of microhomologies between the broken ends of Sμ-Sγ junctions in WT and AID-deficient samples. *χ* ^2^ test was used for significance of IgG CSR structure analysis. **p < 0.01; ***p < 0.001; ****p < 0.0001.

In order to explore the type of recombination occurring in activated B-cells from AID-deficient mice, we used the high-throughput CSRseq method to identify sequences of Sµ–Sγ junctions amplified through long-distance PCR. Sequencing reads showing identical junctions were assembled into clusters which were comparatively quantified for abundance and structure. Experiments using similar inputs (100 ng) of spleen DNA from immunized mice scored much more abundant CSR clusters in WT animals than in AID-deficient mice (mean 569 ± 282 instead of 32 ± 29, *p* = 0.0001), but CSR junctions were thus still clearly detectable and diversified in the latter (**Figure 3A**, top). The CSR defect in AID-deficient B-cells is thus incomplete, and CSR detected in such polyclonal cells remains diversified in terms of breakpoint positions, suggesting that it does not correspond to rare accidental breaks.

We also had the opportunity to analyze DNA from one tonsil and three peripheral blood samples from four immunodeficient patients with a class-switching defect involving biallelic germline mutations located upstream or within the AICDA exon 3 which encodes the catalytic domain of AID. Similar to AID deficient-mice and although in lower abundance than in tonsil DNA from an AID-proficient control, diversified Sμ–Sγ junction sequences were detectable in all four patients (**Figure 3A**, bottom).

The structures of CSR DNA junctions in lymphoid tissues from AID-deficient mice and patients were compared with regards to repair and the relative occurrence of either flush junctions or junctions revealing short insertions or microhomologies between both DNA ends. In both mice (**Figure 3B**, left) and humans (**Figure 3B**, right), junctions characterized in AID-deficient conditions revealed a lower occurrence of flush junctions which directly corresponded to the ligation of blunt ends and an increased occurrence of microhomology between both ends. Sequences from all reads, including junctions and their detailed analysis using CSreport, have been deposited on GEO (GSE183034); examples of junctions obtained in human patients and controls are provided in **Supplementary Table S2**.

**Figure 4:**
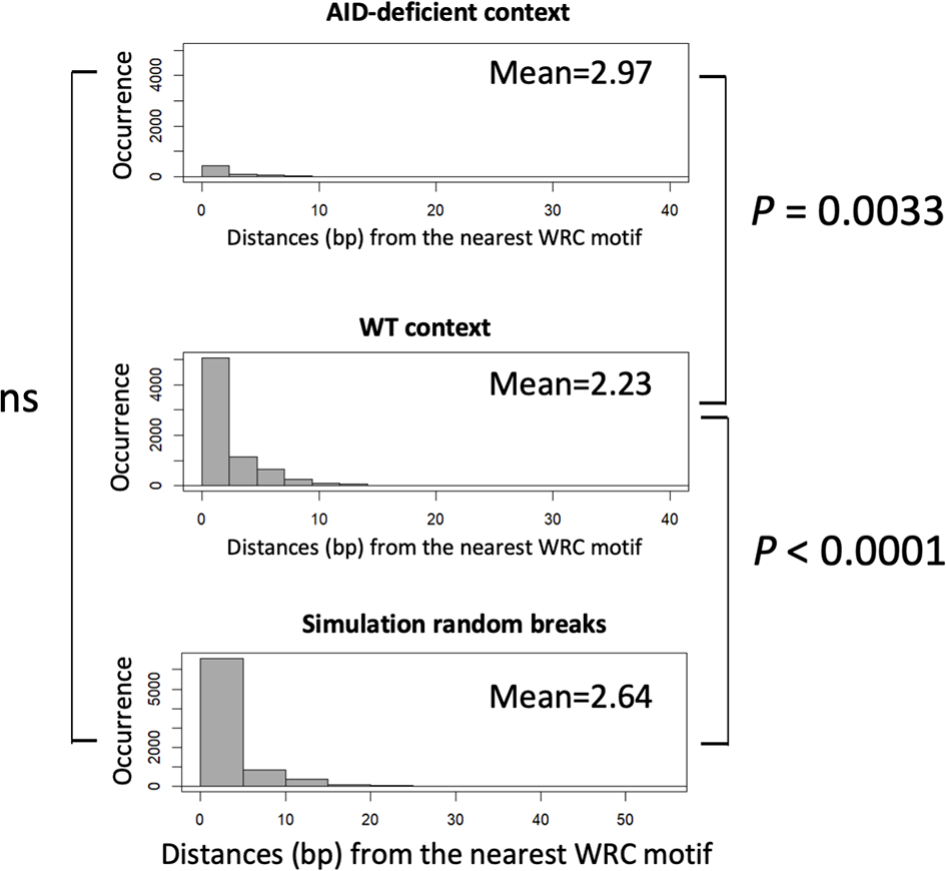
Comparison of distances between CSR breaks and nearest activation-induced deaminase (AID) target sites. Distances between the position of DNA breaks and nearby WRCY sites were scored in either junctions from wild type B-cells, AID-deficient B-cells, or a control reference distribution of random breaks (4,000 junctions). An approximate permutation distribution test built from 100,000 random permutations was performed. ns, non significant.

The examination of junction sequences also determined the distance between the positions of DNA breaks and sites corresponding to classical AID target sites. This distance was significantly increased in junctions from AID-deficient mice compared to wild-type mice (mean 2.97 *vs.* 2.23 nt, *p* < 0.01), showing that DNA breaks were not focused on WRCY motifs but, more probably, randomly affected the fragile portions of S regions (**Figure 4**). Indeed we also compared the distance between DNA break positions in a WT context and in *in silico* simulated events (*i.e*., DNA breaks randomly simulated within the *Sµ* region), and we observed that the WT pattern of breaks differed from the simulated random distribution (*p* < 0.0001). By contrast, the AID-deficient pattern and the simulated random breaks did not significantly differ.

That the CSR defect in AID-deficient B-cells is only incomplete, both in AID-deficient mice and AID-deficient patients, is thus confirmed at the gene level by the occurrence of DNA junctions ligating the usual target regions of CSR, Sμ; and Sγ, but with a random pattern of breaks and an altered pattern of DNA repair suggesting the increased usage of microhomology-mediated end-joining.

**Figure 5:**
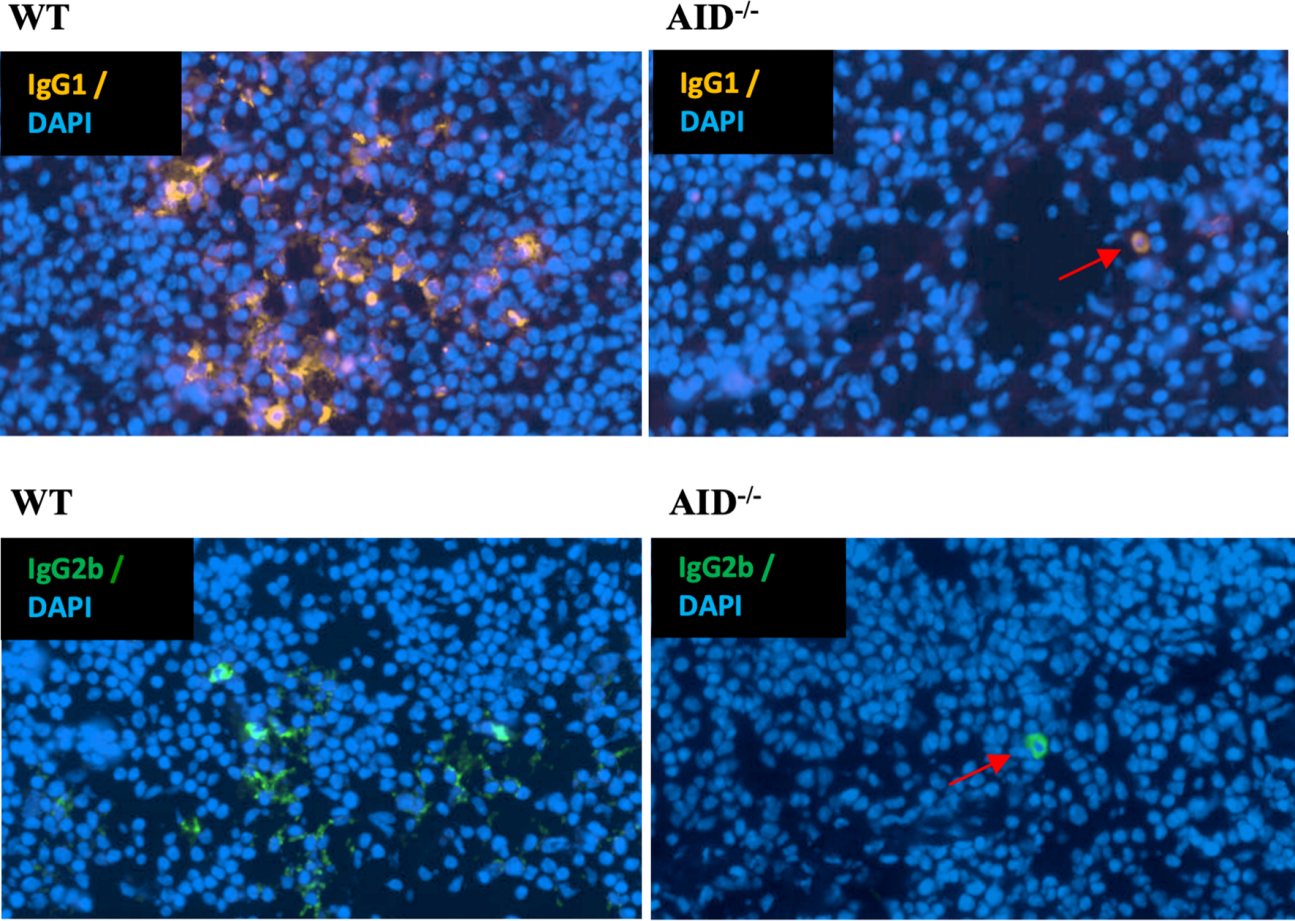
Identification of rare cells producing class-switched Ig in spleens from immunized animals. Representative microscopy fields from slides of spleen tissues obtained from either WT (left) or AID-deficient (right) immunized mice, stained either for mouse IgG1 (top) or IgG2b (bottom). Red arrows indicate rare switched plasma cells in spleen tissue from an AID-deficient mouse.

Finally, in order to check whether the rarely occurring CSR persisting in AID-deficient B lymphocytes might reach a sufficient level for the detection of class-switched cells in lymphoid tissues, we explored the presence of plasma cells producing IgG1 or IgG2b in spleen sections from immunized AID-deficient mice by conventional immunohistochemistry. In agreement with the presence of secreted IgG in serum and of Sμ–Sγ junctions in lymphoid tissue DNA, cells staining for intracellular IgG and with the typical aspect of plasma cells were readily identified in such conditions (**Figure 5**).

## Discussion

The S regions harbor a repetitive structure favorable for clustered AID-mediated DNA lesions and frequent occurrence of close single-strand gaps on opposite strands leading to staggered DSBs. Their structure is unique in terms of primary sequence, architectural organization, and chromatin marks. We hypothesized that such structures might, by themselves, promote DSBs even in the absence of AID lesions, either due to co-transcriptional R-loops and/or due to repeated and overlapping motifs which may favor DNA polymerase slipping during replication and result in transient single-stranded structures. However, AID deficiency is classically considered to abrogate CSR.

This paradigm might, however, overlook a residual level of class-switched Ig produced even in the absence of AID, and, noticeably, the initial report of AID knock-out mice in fact mentioned low but still detectable levels of serum IgG1 and IgG2a (around 1 µg/ml) (22). In some human patients, such as patient P4 in the study by Revy et al. (2) or patient P6 in the current report, low serum IgG also remained detectable at 0.4 and 0.1 mg/ml, respectively. This is especially intriguing in such patients affected with mutations which truncate AID upstream or within its catalytic domain: our patient P6 notably carried a homozygous nonsense mutation as early as codon 22 of the AID coding sequence.

To explore the hypothesis that a low rate of CSR junctions could be dynamically induced in activated B-cells in the absence of AID, we used sensitive methods to measure residual CSR in AID-deficient B-cells.

IgG production by AID-deficient mice was indeed detected *in vivo* and strongly increased after immunization, with plasma cells producing switched IgG detectable in tissues from immunized animals. We also observed that these switched Ig could be detected as Ag-specific antibodies in low amounts but dynamically increasing after immunization, *i.e.*, with kinetics resembling normal immune humoral responses and not bystander production after random recombination.

Characterization of switch junctions at the DNA level confirmed the occurrence of DNA breaks within the classical target Sμ and Sγ regions and also revealed an altered pattern of repair, suggesting the lesser involvement of non-homologous end-joining, rather yielding flush junctions, and with increased involvement of non-classical alternate end-joining, which is supported by short microhomologies between DNA ends.

Whether such breaks in the S regions, responsible for basal CSR, constitute an intrinsic property of S regions by behaving as fragile sites will remain to be determined. Noticeably, the process remains inducible, showing that such an intrinsic “fragility” is not simply related to the DNA structure but also needs B-cell activation, germline transcription of S regions, and all of the processes usually considered to facilitate the recruitment and processivity of AID for mediating DNA lesions. In this regard, the G4 richness of S regions might expose DNA to breaks, as it was shown to favor non-B DNA structures and to confer transcription-dependent instability to S regions transferred in yeast (23). The active transcription of S-regions in Ag-stimulated B-cells is thus likely to facilitate transcription–replication conflicts (TRCs) and the occurrence of single-strand DNA breaks at the positions of the G4 DNA and R-loops, notably due to the activity of helicases and of endonucleases like XPG and CtIP or of the exonuclease Exo1, all previously reported to promote the occurrence of breaks at R-loops or TRCs (24–27).

The chromosomal context of S regions might also intrinsically expose them to AID-independent breaks. Prior to any AID activity, the S regions are under the control of their upstream cytokine-dependent germline promoters and of the *cis*-acting 3′RR superenhancer (28–31). This promotes major dynamic changes, marked by germline S region transcription, a modified histone mark landscape, and 3D remodeling including local co-transcriptional R-loops and the long-range cohesin-dependent loop extrusion process, which is driven by IgH promoters and the 3′RR superenhancer and finally juxtaposes distant transcribed S regions (30, 32, 33).

Although AID is considered mandatory for CSR and SHM, it is also questionable whether other cytidine deaminases of the APOBEC family might target DNA at low levels and not only RNA.

Low levels of DNA cytidine deamination have been described for APOBEC 3 and APOBEC1 (34, 35), and although their activity has never been demonstrated in Ig genes nor shown to induce DSBs, it is conceivable that, as for AID, staggered single-strand breaks induced after deamination in S regions might promote DSBs and CSR.

AID deamination occurs in the G1 phase, where 53BP1 and γH2AX protect SSBs and lead to repair through classical NHEJ (36, 37). Beyond G1, RPA associates with unrepaired ends in an ATM-dependent manner and favors repair by micro-homology-dependent alternate NHEJ (A-NHEJ). Finally, DNA breaks persisting in the S/G2 phase recruit higher amounts of Rad51 and are preferentially repaired by error-free homologous recombination (38). Increased repair through A-NHEJ is thus an indication that AID-independent breaks occur later in the cell cycle. Such breaks might also lack the intervention of AID and of downstream factors such as UNG in promoting synapsis and repair (39). While IgH breaks joined to *c-myc* were previously reported in AID-deficient mice after pristane-induced lymphomagenesis, they were, however, not shown to affect the S regions (40). Noticeably, while AID strongly contributes to DNA breaks with legitimate repair and CSR, it also supports illegitimate repair with non-Ig loci during GC-derived lymphomagenesis (41).

Our work is reminiscent of previous studies where experimental genomic breaks elicited by nucleases identified AID-independent hotspots as partners for repair, which included Sµ and Sγ regions in activated B-cells (42). The AID defects result in severe immunodeficiency, and its role comes on top of several processes shaping Ig loci as optimal substrates for AID lesions and occurrence of DNA breaks. Even in the absence of AID, it is thus not unexpected that some accessibility to breaks remains. AID has multiple interactions with factors regulating its nuclear location and activity. This includes nuclear factors involved in transcription elongation and pausing, RNA splicing and degradation, DNA repair, heterochromatin-specific factors, and components of nucleoli: eEF1A, the Spt5/RNAPII/PAF complex, CTNNBL1, GANP, the nascent RNA-degrading exosome complex, RNF126, REG-γ, RPA (bound to pSer38-AID), and the heterochromatin factors Kap/HP1 (binding H3K9me3 on Sµ), nucleolin, nucleophosmin, and 14-3-3 (binding WRCY repeats and helping recruit CSR co-factors together with AID) (43). All the functional roles of AID interactions with other partners have not yet been elucidated. While some factors interact with AID after the occurrence of SSBs, others are recruited on Ig genes prior to AID. This is notably the case of factors binding the structural features of transcribed S regions loaded with paused RNAPII (Spt5/RNAPII/PAF complex, 14-3-3, GANP, the nascent RNA-degrading exosome or the heterochromatin factors Kap/HP1, RPA, *etc.*). Whether such factors might, by themselves, contribute to the occurrence of DNA lesions independently of AID will remain to be determined.

## Data Availability Statement

The datasets presented in this study can be found in online repositories. The names of the repository/repositories and accession number(s) can be found below: (https://www.ncbi.nlm.nih.gov/), GEO Accession GSE183034.

## Ethics Statement

The studies involving human participants were reviewed and approved by Comité de protection des personnes Ile de France. Written informed consent to participate in this study was provided by the participants’ legal guardian/next of kin. The animal study was reviewed and approved by Ministère de la recherche, l’enseignement supérieur et l’innovation (APAFIS#16689-2018091017202113 v3).

## Author Contributions

ID, ZD, and BW participated in investigation, methodology, and writing of the original draft (supporting). BL participated in data curation and writing of the original draft (supporting). FJ and BB participated in methodology. PD participated in formal analysis. JM participated in writing—review. SK, AF, and AD participated in providing samples from patients and writing of the original draft (supporting). SN participated in data curation and formal analysis. MC led the conceptualization, data curation, funding acquisition, writing of the original draft, and review and editing as well as participated in formal analysis. All authors contributed to the article and approved the submitted version.

## Funding

This work was supported by grants from Agence Nationale de la Recherche (grants ANR-16-CE15-0019-01 and 18-CE18-0022-02) and Fondation ARC (grant PGA1 RF20180207070).

## Conflict of Interest

The authors declare that the research was conducted in the absence of any commercial or financial relationships that could be construed as a potential conflict of interest.

## Publisher’s Note

All claims expressed in this article are solely those of the authors and do not necessarily represent those of their affiliated organizations, or those of the publisher, the editors and the reviewers. Any product that may be evaluated in this article, or claim that may be made by its manufacturer, is not guaranteed or endorsed by the publisher.
