# Supplementary material for: UnAIDed Class Switching in Activated B-Cells Reveals Intrinsic Features of a Self-Cleaving IgH Locus
Source: Front Immunol. 2021 Oct 28;12:737427. doi: 10.3389/fimmu.2021.737427 (PMC8581400; doi:10.3389/fimmu.2021.737427)
Supplement: Supplementary file 1 [file DataSheet_1.pdf]

LEGENDS TO SUPPLEMENTAL INFORMATION

Supplemental Figure 1: AICDA gene alterations in patients P3, P4, P5 and P6.

The coding region of exons is in red. Exon 3 encodes the catalytic domain of the AID enzyme.

Supplemental Figure 2: Validation of IgG and IgA-specific ELISA.

Mouse monoclonal antibodies were obtained from a commercial source (IgM standard, Southern Biotechnologies) and from the French National Blood Center (clones BHS17, AY144 and E11) to test the specificity of anti-IgG and anti-IgA antibodies used for ELISA in this study. Serum from a naive AID-deficient mouse (mouse 9990) with spontaneously high IgM and not producing detectable IgG or IgA at Day 0 provided at internal control showing that the rise of IgG and IgA at Day 17 after SRBC immunization was not a cross-reaction with polyclonal IgM.

Supplemental Figure 3: IgM levels do not correlate with IgG or IgA levels.

Serum IgM, IgG and IgA concentrations were determined by ELISA. Each point represents one AID-deficient mouse, before or after SRBC immunization. The linear regression shows that there is no correlation between IgM and IgA concentrations (top), or between IgM and IgG concentrations (bottom) (R^2 <0.3 in both cases).

Supplemental Table 1:

Genomic coordinates used for aligning switch junctions from mouse and human samples are indicated.

Supplemental Table 2:

Example of junction sequences. For each sequence read including a Sμ-Sγ1 junction, positions of both DNA breaks in Sμ and Sγ are given by reference to chromosome GRCh38:14 (start at 105863000:1, reverse orientation). The fifty bases-long sequence centered onto the junction is shown. **Sμ is in bold,** Sγ is underlined, **microhomologies are bold and underlined**, N stand for an inserted nucleotide.

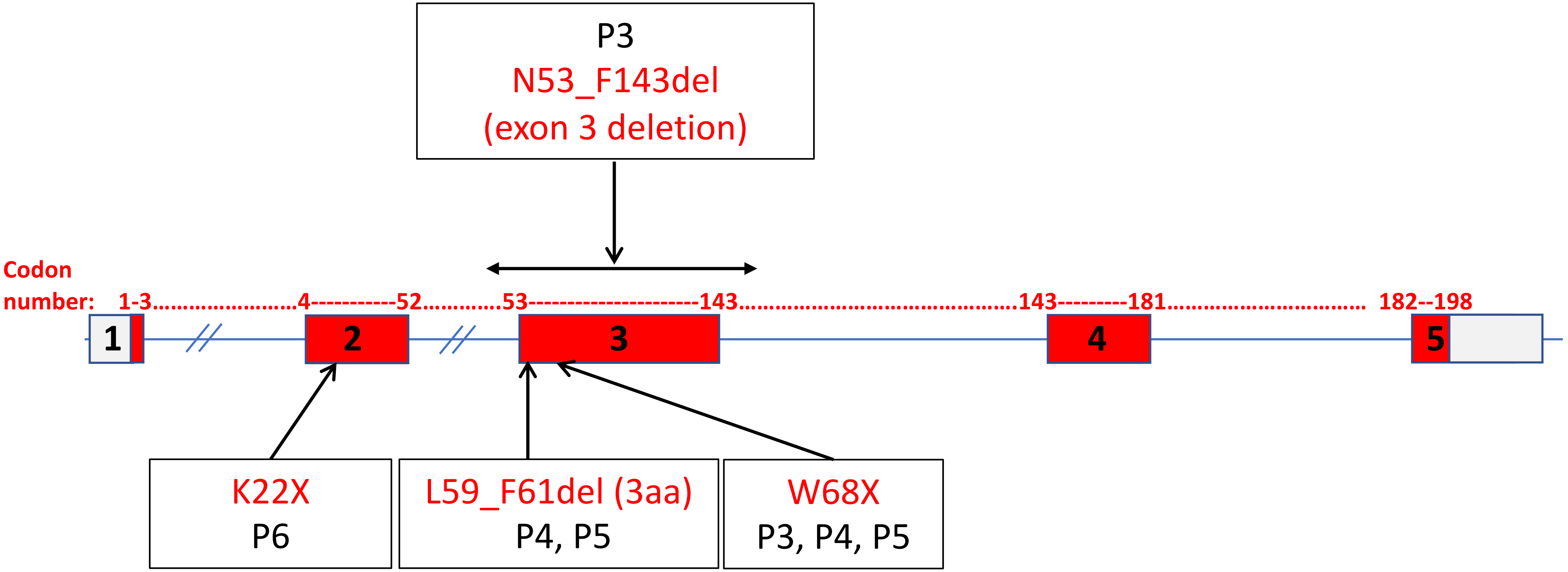

Mutations in AID-deficient patients:

- Patient P3 : W68X termination / N53_F143del (exon 3)
- P4 and P5 : W68X termination / L59_F61del (exon 3)
- P6: homozygous K22X termination (exon 2)

Supplemental Figure 1

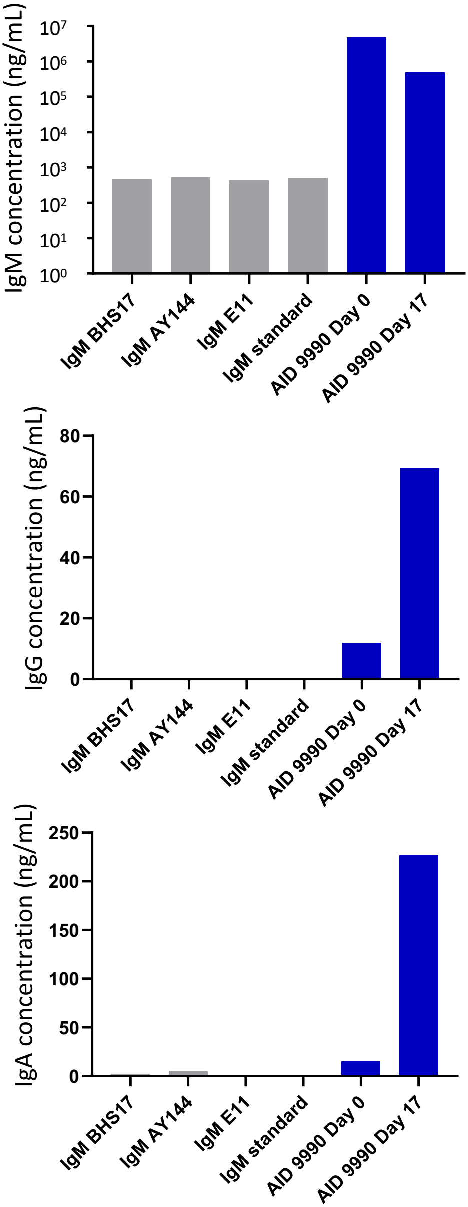

Supplemental Figure 2

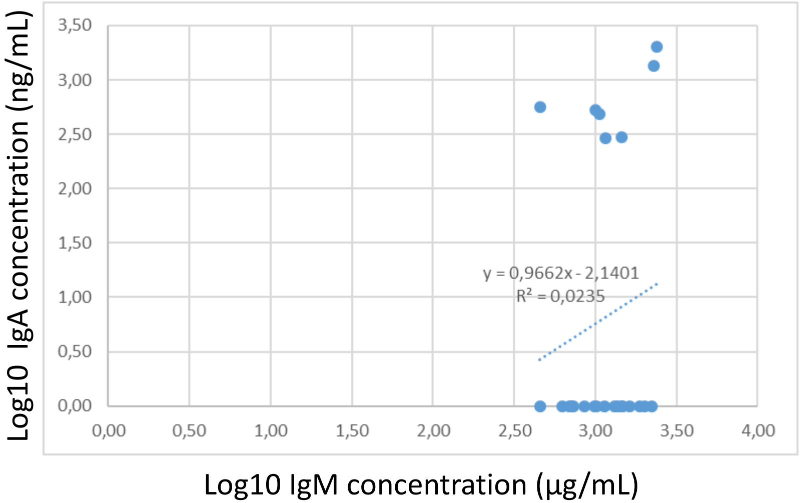

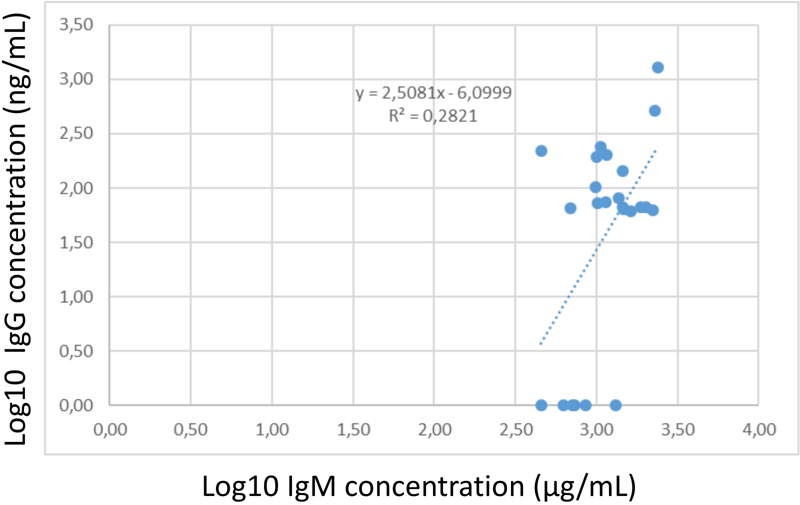

Supplemental Figure 3

Mouse sequence alignments:

IgH constant gene cluster 218,000 bp sequence considered for analysis with CSReport algorithm:
Mus musculus, chromosome 12, GRCm38.p3 C57BL/6J, gi|372099098: c113208001 (end)-113426000 (start)

Switch μ	0	3270
Switch γ3	59768	64768
Switch γ1	86477	95477
Switch γ2b	111067	118067
Switch γ2c	129068	137068
Switch ε	147752	152752
Switch α	158764	165764

Human sequence alignments:

IgH constant gene cluster 313,000 bp sequence considered for analysis with CSReport algorithm:
Homo sapiens, chromosome 14, GRCh38:14:c105550001 (end):105863000 (start)

Switch μ	2253	6781
Switch γ3	87094	91594
Switch γ1	115553	119928
Switch α1	151040	154334
Switch γ2	214709	218209
Switch γ4	232433	236933
Switch ε	257290	261271
Switch α2	271075	274604

Supplemental Table 1: Genomic coordinates used for aligning switch junctions from mouse and human samples are indicated.

Examples of Sμ-Sγ1 junctions from AID-/- patients

(patients P3 and P5 with complete homozygous absence the AID catalytic domain)

P3	
>2491-119076-mHomo-2	**GTGCAGGAACCCGGCAATGAGATGG**GAGGAGGTGGCTGGTGGCCGAGAA
>4262-119823-mHomo-5	**CTGATTTGGGCTGGGTTGAGCAGAC**ACTGGACGCTGAACCTCGCGGACAG
>3010-119769-mHomo-2	**GCTGAGCTGGGCTGGGCTGAGCTGG**CTAAGGTGACGCAGGTGGCGCCAGC
>2812-118856-mHomo-4	**TGAGCTGGGCTGGGCTGGGCTGGGC**GCTTAGCCTCCTCAGAGGTCAGGGG
>2521-118278-Blunt-0	**GCTGAGACAAGCAGGTCTGGTGGGC**GGCAGGACGAGCAGGGGACAGGCAC
>2520-116813-mHomo-6	**AGCTGAGACAAGCAGGTCTGGTGGG**AAGCATGGGGAGCAGGTGGGCAGCC
>2520-116654-mHomo-8	**AGCTGAGACAAGCAGGTCTGGTGGG**AGGCGGGAGGAACAGGGGGCAGCTC
>2516-118881-mHomo-2	**CTTTAGCTGAGACAAGCAGGTCTGG**GGCACACCCCACCTGCAGTGGGACT
>2513-117954-Blunt-0	**TGGCTTTAGCTGAGACAAGCAGGTC**CAGGTGGGGGGCAGGAGGAGCAGGG
>2511-118287-mHomo-6	**GATGGCTTTAGCTGAGACAAGCAGG**GGACAGGCACTAGAGCTCAGGGCAA
P5	
>2474-117725-mHomo-8	**TGGAAGCCAGCCTGGCTGTGCAGGA**GGAGCAGGGGGCAGGCACTGGAGCT
>4912-118286-mHomo-4	**TGAGCCGGACTGGGTTGGGCTGAGC**AGGGGACAGGCACTAGAGCTCAGGG
>2997-118523-mHomo-1	**TGGGCTGGGCTGCGCTGAGCTGGGC**AGGGCTGAGCTCTCAGGCAGGGGCA
>2512-117350-mHomo-7	**ATGGCTTTAGCTGAGACAAGCAGGT**GGGCAGCCCCTGGAGCTCAGAGAGC
>2512-116970-mHomo-6	**ATGGCTTTAGCTGAGACAAGCAGGT**GGGAGGCAGAATAAGCAGGGGACAG
>2466-116496-mHomo-5	**ACAAAAGATGGAAGCCAGCCTGGCT**AGTGGGAGATGGCAAGCAGGGGGC

Examples of Sμ-Sγ1 junctions from a healthy human control

>2646-119430-Blunt-0	**GCTGAGCTGAGCTGAACTGGGCTGA**CCACGCTGGGGCGGCAGGGTCAGGG
>2472-118763-mHomo-4	**GATGGAAGCCAGCCTGGCTGTGCAG**AGTCAGCCAGAGCCTGTGGTGCAGG
>2464-118345-mHomo-2	**AGACAAAAGATGGAAGCCAGCCTGG**AGGCATCACTCAGCTCCTAGACTTT
>2489-118566-Blunt-0	**CTGTGCAGGAACCCGGCAATGAGAT**AACGTGTGCTTGAGACCAGGAGGGT
>2455-119150-mHomo-4	**AGTAGAGGGAGACAAAAGATGGAAG**TAATGCAGGACACTGGGAGGCAGGG
>3478-118971-Blunt-0	**GAGCTGAGCTTAGCTGGGCTGAGCT**CCATGCAGGGTGGGGAGGGCCAAGG
>2667-119677-mHomo-2	**CTGAGTTGAACTGGGTTGAGCTGAG**GGGTGCCTGCAGAAGGGCACCACAG
>2692-117317-mHomo-4	**CTGAGCTGAGCTGGGCTAAGTTGCA**GGTGAGCAGGGCCGGTGGGAAGCAG
>2526-118312-Blunt-0	**GACAAGCAGGTCTGGTGGGCTGACC**GGGCAAGGCAGCCACAGGTGAGCAG
>2487-118505-mHomo-1	**GGCTGTGCAGGAACCCGGCAATGAG**GAAGGCGTTCCAGGGCTCAGGGCTG
>2621-118522-Blunt-0	**CCTAGACTAAACAAGGCTGAACTGG**TCAGGGCTGAGCTCTCAGGCAGGGG
>2686-118513-Insert-1	**GCTGAGCTGAGCTGAGCTGGGCTAA**NTTCCAGGGCTCAGGGCTGAGCTCT
>2631-119304-mHomo-5	**ACAAGGCTGAACTGGGCTGAGCTGA**AGGGCGCCGACCCTATGCAGTGTCT
>2684-119725-mHomo-1	**GAGCTGAGCTGAGCTGAGCTGGGCT**CTGGGGCAGGCCGGGCCTGACCTTG
>2587-118330-Blunt-0	**ATGGACTCAGATGGGCAAAACTGAC**TGAGCAGGGCTGGTGGGAGGCATCA
>2711-118904-mHomo-1	**GTTGCACCAGGTGAGCTGAGCTGAG**ACTCCAGGGCCACTGGGCCAGCGGC

(**Sμ is in bold,** Sγ is underlined, **microhomologies are bold and underlined,**
N stand for an inserted nucleotide).

Supplemental Table 2. Example of junction sequences.
